# Synergistic grasp analysis: A cross-sectional exploration using a multi-sensory data glove

**DOI:** 10.1017/wtc.2024.25

**Published:** 2025-01-23

**Authors:** Subhash Pratap, Kazuaki Ito, Shyamanta M. Hazarika

**Affiliations:** 1 Biomimetic Robotics and AI Lab, Mechanical Engineering, IIT Guwahati, Guwahati, Assam, India; 2 Department of Intelligent Mechanical Engineering, Gifu University, Gifu, Japan

**Keywords:** grasp, synergies, multi-sensory, data glove, human-centered computing

## Abstract

This paper investigates hand grasping, a fundamental activity in daily living, by examining the forces and postures involved in the lift-and-hold phases of grasping. We introduce a novel multi-sensory data glove, integrated with resistive flex sensors and capacitive force sensors, to measure the intricate dynamics of hand movement. The study engaged five subjects to capture a comprehensive dataset that includes contact forces at the fingertips and joint angles, furnishing a detailed portrayal of grasp mechanics. Focusing on grasp synergies, our analysis delved into the quantitative relationships between the correlated forces among the fingers. By manipulating one variable at a time—either the object or the subject—our cross-sectional approach yields rich insights into the nature of grasp forces and angles. The correlation coefficients for finger pairs presented median values ranging from 0.5 to nearly 0.9, indicating varying degrees of inter-finger coordination, with the thumb-index and index-middle pairs exhibiting particularly high synergy. The findings, depicted through spider charts and correlation coefficients, reveal significant patterns of cooperative finger behavior. These insights are crucial for the advancement of hand mechanics understanding and have profound implications for the development of assistive technologies and rehabilitation devices.

## 1. Introduction

The human hand is a versatile and complex system with dexterous manipulation capabilities. Grasping objects to interact with the environment is accomplished using the hand. Studies have estimated that during a typical 8-hour workday, an average person performs between 4000 to 5000 grip changes (Riddle et al., 2020). “A grasp is every static hand pose with which an object can be held securely with one hand” (Feix et al., 2015). Grasp-hold-release tasks are undoubtedly among the most common in various DLAs (Hussain et al., 2018). Hand grasp patterns require complex coordination. The term “synergy,” derived from the Greek word “synergia,” signifies the concept of elements working together towards a common objective (Sancho-Bru et al., 2011).

**Figure 1.**
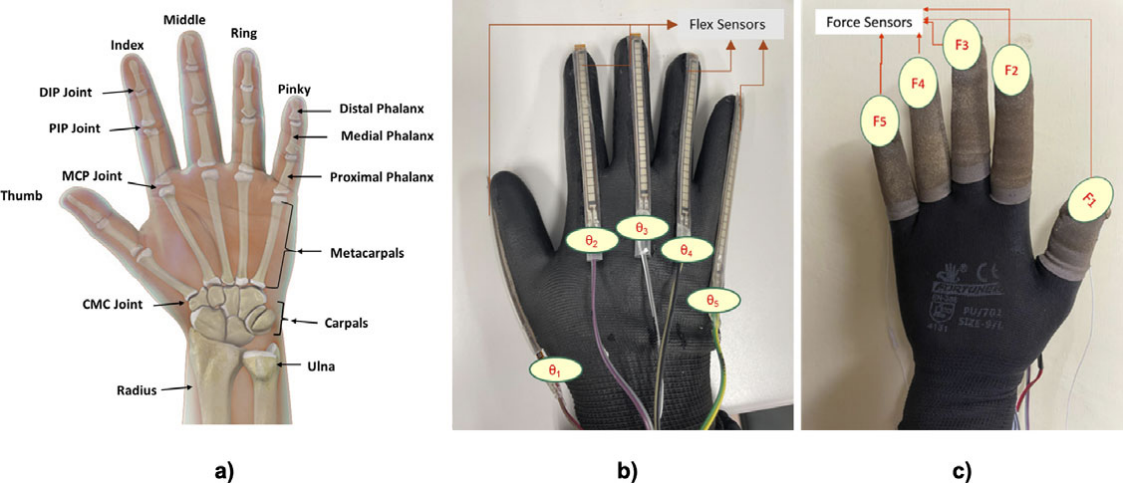
(a) The human hand anatomy (b) dorsal side of the data glove with flex sensors (c) palmer side of the data glove with force sensors.

Figure 1 (a) displays the typical structure of a human hand, including the thumb, index, middle, ring, and little fingers. A human hand comprises 27 bones: five distal phalanges, four middle phalanges, five proximal phalanges, five metacarpals, and eight carpals. Each of the five fingers, including the thumb, is composed of segments: two for the thumb (proximal and distal phalanges) and three for the other fingers (proximal phalanx, middle phalanx, and distal phalanx). The index, middle, ring, and little fingers include the metacarpal-phalangeal (MCP) joints between the metacarpal and proximal phalange bones and the proximal and distal interphalangeal (PIP and DIP) joints that separate the phalangeal bones. The thumb has MCP and interphalangeal (IP) joints. In terms of degrees of freedom (DOFs), each finger has four (two at MCP, one at PIP, and one at DIP), the thumb has three (two at MCP and one at IP), the wrist has two, and the carpometacarpal (CMC) joint has two (Chen and Naidu, 2013). As a result of its kinematic structure, our hands and fingers have a high degree of dexterity and are capable of performing a variety of fine motor movements which allow us to perform DLAs (Sancho-Bru et al., 2011). However, only a reduced number of manipulation postures are consistently used in our day-to-day lives.

Understanding the way humans grasp objects, with insight into the kinematic implications and limitations associated with each grasp and knowing together with common use patterns are important in numerous domains ranging from medicine, rehabilitation, psychology, product design, and many others. Hand grasps necessitate intricate coordination (Santello et al., 2016). This is particularly relevant to the pre-grasping phase, which involves shaping the hand before it comes into direct contact with the object to be grasped. These synergies can be described as a spatial configuration or a “primitive” hand shape that is commonly shared across various tasks. Human grasping actions involved in the activities of daily living is a complex process that utilizes the hand’s multiple degrees of freedom and takes advantage of redundancy in both movement and muscle utilization. These coordinated movements, known as grasp synergies, are crucial for efficient hand function. Deepening our knowledge of the dynamic aspects of these grasping actions can significantly enhance the application of these principles to the design and function of robotic hands. The exploration and understanding of these grasp synergies, particularly in the context of the kinematics of hand positioning, continue to be a vigorously pursued area of research, as highlighted by Starke et al. (Starke et al., n.d.).

Despite the hand’s vast potential for manipulation, it typically employs a finite set of interaction modes with objects, commonly categorized as “grasp types.” These types serve as a framework for characterizing hand utilization. Numerous classification systems for these grasps have been presented throughout scholarly works (Cutkosky and Wright, 1986; Bullock et al., 2013). One of the pioneering figures in this area, Schlesinger, delineated six fundamental grasping patterns: tip, spherical, hook, palmar, lateral, and cylindrical. This early classification underscored that the positioning of the hand is influenced by the object’s physical attributes (Schlesinger, 1919). Building on this, Napier et al. posited that the nature of the task at hand also dictates the type of grasp (Napier, 1956). Expanding on these concepts, Cutkosky et al. (Cutkosky and Wright, 1986) developed a more comprehensive taxonomy that considers the decisions leading to a grasp and the object’s geometry. The Yale-CMU-Berkeley (YCB) Object and Model Set (Calli et al., 2015), designed for robotic research, comprises 26 tasks involving eight key human grasp types and two non-grasping postures, spanning food, kitchen, tools, shapes, and task-specific items. This diverse collection facilitates replicable studies in robotic manipulation and human-robot interaction. Accurately describing and quantifying the forces involved in grasping remains a complex endeavor (Starke et al., n.d.). The intricate biomechanical structure and neural mechanisms of the human hand present significant challenges in comprehending: (a) the control mechanisms governing the synchronized motion of the fingers, and (b) the necessary grasp forces for handling an extensive array of objects in daily living activities (DLAs), which include actions from grasping with multiple digits to the movement of individual fingers. Grasp synergy refers to a basic spatial configuration of the hand, frequently seen across various tasks. The intricate biomechanical and neurological makeup of hand joints leads to challenging questions about the control processes that coordinate finger movements and the grip strength required to handle diverse objects in daily living activities (DLAs). This complexity encompasses numerous tasks, from holding items with multiple fingers to the precise motor control of each individual finger. The exploration of grasp synergies, particularly in relation to hand postures and forces, is a rapidly evolving field of study. Santello et al. (Santello et al., 2016) posited that a limited number of postural synergies govern hand posture during grasping, effectively simplifying the complexity of joint angle variability into a more manageable, lower-dimensional framework. This concept has been widely adopted in neuroscience, forming the basis for various theoretical models, experimental designs, and analytical methods aimed at understanding the neural mechanisms governing hand movement, with significant implications for neuro-rehabilitation. Olikkal et al. further contributed by demonstrating that the integration of kinematic data with muscle synergy information through data fusion techniques can significantly enhance the accuracy of movement replication, highlighting its utility in improving prosthetic and exoskeleton controls (Olikkal et al., 2022). Burns et al. (Burns et al., 2019) introduced a control system reliant on spatiotemporal synergies for assistive hand devices, achieving enhanced grasping dexterity with reduced computational demands. Their research offers valuable guidance on selecting the appropriate number of synergies for control system design, balancing controller accuracy and system complexity.

Measurement of hand kinematics and dynamics is crucial for assessing the human hands’ synergy in terms of grasp postures and forces. Traditional methods involve using mechanical goniometers placed on joints to measure movement range. Mechanical goniometers lack precision and cannot offer dynamic measurements (Mohan et al., 2018). An alternative approach is using sensors, often integrated into gloves, to monitor hand movements, providing a more practical and dynamic solution. Data gloves have emerged as valuable tools across a range of uses, such as evaluating hand functions and aiding in rehabilitation efforts. Zheng et al. introduced an electronic glove designed for the analysis of safe handling by bionic devices (Zheng et al., 2021). Similarly, Nassour et al. developed a sensor-equipped glove capable of capturing hand movements, which aids in duplicating these motions and calculating hand joint angles (Nassour et al., 2020).

In this study, we present an investigation into the kinematics and dynamics of hand grasping, specifically focusing on the critical lift and hold stages that are vital for performing everyday activities. This research explores the concept of grasp synergies within the realms of force and posture. Force synergies are conceptualized based on the forces exerted at the points of contact, drawing from the observed relationships among the forces generated by the fingers. To accurately capture these grasp postures and forces, we developed a multisensory data glove equipped with resistive flex sensors for posture detection and capacitive sensors for measuring force. This study involved five participants, each executing five distinct types of grasps commonly encountered in daily living activities. Using the multisensory glove, we were able to record the contact forces at five fingertip locations and corresponding joint angles. Through this approach, our research offers a detailed examination of the hand grasping mechanisms, facilitated by the use of an innovative multisensory data glove. This involved a cross-sectional analysis focusing on the variations in grasp forces among different subjects and objects. Our approach was to methodically observe how these force values change under varying conditions. By keeping one variable constant—either the object or the subject—and varying the other, we aim to uncover deeper insights into the dynamics of grasp forces and finger-bending angles. This method allows us to observe how these factors differ among various individuals and with different objects. Such an analysis is expected to provide valuable information that can enhance our understanding of hand mechanics, particularly in the development of assistive technologies and rehabilitation devices. This approach underscores the complexity and individuality of hand movements, emphasizing the need for personalized solutions in hand rehabilitation and assistive device design. To explain the results related to pose and force are intuitive (or otherwise), we have consistently used the cylindrical grasp as an example for detailed discussion, throughout the results section. This is because the cylindrical grasp represents one of the most commonly used and versatile grasps, encompassing significant contributions from all five fingers. It involves a well-distributed combination of flexion, force, and coordination among the digits, making it a comprehensive and intuitive choice for illustrating grasp dynamics. To illustrate the distinct patterns of grasping for different objects, even within the same grasp type, we employed spider charts to represent the grasp synergies. These visual representations are instrumental in highlighting the unique grasp characteristics for each type of grasp. A significant aspect of our study was the analysis of the correlation coefficients between pairs of fingers. This analysis revealed a notable cooperative behavior among the fingers during grasping tasks. Such findings are crucial as they contribute to our understanding of grasp synergy behavior in human hands. This synergy is particularly relevant in the context of designing underactuated devices for hand rehabilitation (Alicea et al., 2021), where understanding the natural coordination between fingers can lead to more effective and intuitive control mechanisms.

## 2. Materials and methods

In this section, we detail the deployment of an advanced multi-sensory data glove, which is outfitted with flex and force sensors. This device is pivotal in accurately capturing the complex dynamics of grasping across various objects used in DLAs. Our study meticulously recorded the exerted fingertip forces and bending angles of the fingers from five participants, yielding a nuanced understanding of hand-grasping mechanics.

### 2.1. Fabrication of the multi-sensory data glove

A glove equipped with sensors has been fabricated to measure movements related to the bending and straightening of fingers, in addition to the forces exerted by the fingertips. This glove incorporates a base layer of a compression glove, to which Finger Tactile Pressure Sensors (FingerTPS, Pressure Profile Systems, based in Los Angeles, CA, USA) and Flex sensors (manufactured by Spectra Symbol in Salt Lake City, UT, USA) are attached along the length of each finger. The arrangement of these sensors is depicted in Figure 1(b) and (c), showing that the force sensors are mounted on the fingertips as caps, with their detection elements precisely positioned for optimal force measurement. The collection of accurate force metrics in real-time is facilitated through the use of Pressure Profile Systems’ Chameleon Software. Located on the glove’s dorsal side, the Flex sensors are encased in slim tubes, allowing them to align with and respond to the fingers’ movements. These sensors seamlessly conform to changes in finger posture and are connected to a control board, which is configured using Arduino software.

#### 2.1.1. Measurement of finger bending angle

**Figure 2.**
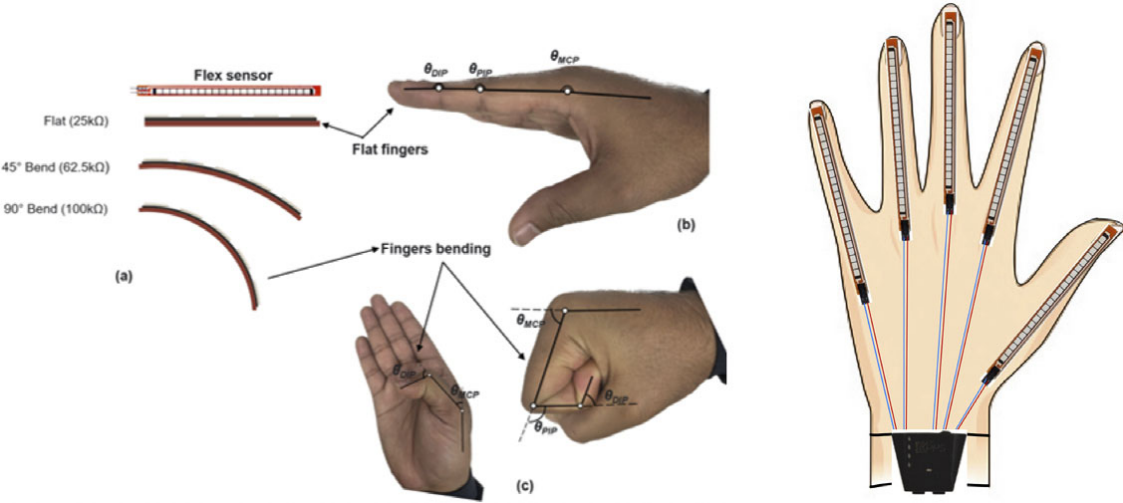
Flexion/extension measurement via flex sensor. (A) Illustration of the resistive flex sensor transitioning from its neutral position *(* 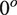 *)* to a bent state *(* 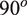 *)*; (B) Initial position of MCP, PIP, and DIP joints in a state of rest (with 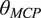 = 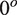 , 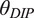 = 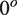 , and 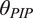 = 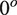 ); and (C) The condition of maximum bending at the MCP, DIP, and PIP joints (D) a Schematic diagram of the flex sensors arrangement over fingers.

Integrating Flex sensors into the glove aims to capture real-time information on the range of motion (ROM) experienced during grasping activities. The characteristics of the Flex sensor, which measures 4.5 inches in length, are outlined in Table 1. Normally, the metacarpophalangeal (MCP) joints function distinctly from the distal interphalangeal (DIP) and proximal interphalangeal (PIP) joints, with the latter two often flexing in unison. It is critical to acknowledge that Flex sensors provide a general measurement of flexion rather than an exact angle of individual finger bends. Consequently, the data captured by these sensors should be regarded as an aggregate flexion angle across the finger’s joints, as depicted in Figure2. This approach allows for a broader understanding of hand movement during grasp execution. The empirical model representing the typical range of motion in human finger movements was outlined in a study by Syed et al. (Ali et al., 2019) (see Figure 2).

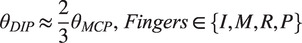




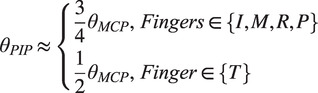

where, T, I, M, R, and P correspond to the Thumb, Index, Middle, Ring, and Pinky fingers, respectively.

**Table 1.** Specification of flex sensor

Parameter	Value
Length	4.5 inch (11.43 cm)
Flat resistance	10 K Ohms
Resistance tolerance	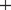 30%
Length	12 cm
Thickness	1 mm
Bend resistance range	60 K to 110 K Ohms
Power rating	0.50 Watts continuous, 1 Watt Peak
Life cycle	> 1 million

Following the methodology established in our previous research (Pratap et al., 2023), Figure 2 illustrates the operational states of a Flex sensor, from its neutral position at an angle of 180  to its fully bent condition at 0  . Each of the five resistive Flex sensors is energized using a consistent 5 V supply. In the sensor’s default state, where it lies flat corresponding to an unflexed position of the MCP, PIP, and DIP joints as shown in Figure 2 (A) and (B), a resistance of 25 kΩ is observed. The resistance value of the sensor escalates as the finger articulates. This variation in resistance is quantified by integrating the Flex sensor with a stable resistor, valued at 47 kΩ, within a voltage divider circuit. The resultant output voltage is indicative of the voltage decrease across the fixed resistor, rather than the Flex sensor itself. The formula for calculating the output from this circuit configuration is provided as follows:

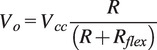

where, 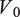 represents the output voltage, 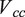 signifies the system voltage, R stands for the pull-down resistor’s value, and 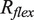 corresponds to the resistance of the flex sensor.

The sensor calibration process involved obtaining resistance values at 0 degrees and 90 degrees. Calibration was achieved by utilizing the map function to correlate sensor resistance with angle values within this range. The angle data was stored in a variable using the flex function, which calculates resistance based on the sensor’s voltage. As the flex sensor bends, its resistance changes, allowing us to map these resistance changes to specific angles. It is worth noting that after several trials, the sensor’s resistance may return to its normal zero state. To address this, we incorporated the corresponding resistance value for zero bending angles directly into the Arduino code, ensuring accurate calibration.

The calibration helps to account for any variability in sensor performance and individual user differences. The calibration procedure for flex sensors in the glove begins with aligning each sensor using a goniometer, establishing a zero-degree angle as the baseline for optimal accuracy. The sensors are incrementally bent to specific angles, up to 90 degrees, while recording the resistance at each step. This process creates a clear correlation between the bend angle and sensor output changes. The error associated with this aggregate measurement can vary based on the individual flexion patterns of the joints. Based on calibration characteristics shown in Table 2, uncertainty values range from 0.55% for the thumb to 5.37% for the index finger, which provides an estimate of the potential error. However, this error is particularly noticeable when different joints within a finger bend unevenly. By using the goniometer for calibration, the process ensured precise and reliable measurements across the full range of motion for each finger, minimizing errors in detecting flexion angles during grasping tasks.

**Table 2.** Calibration characteristics of flex sensors

Finger	Sensitivity (k 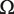 /  )	Resolution (  )	Uncertainty (%)
Thumb	93.11	0.49	0.55
Index	74.11	4.83	5.37
Middle	73.67	1.72	1.91
Ring	98.11	4.15	4.61
Pinky	103.67	2.12	2.36

#### 2.1.2. Tactile force measurement

**Figure 3.**
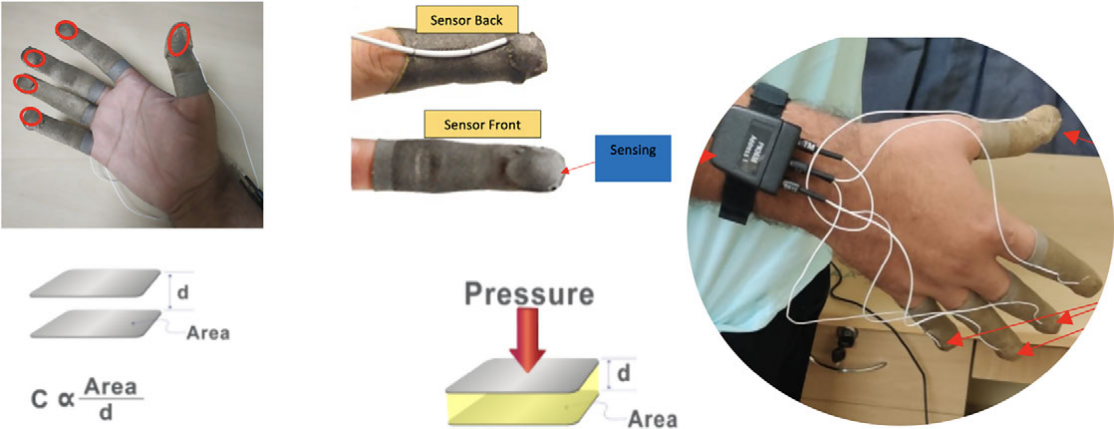
The finger TPS force sensor setup along with the sensing elements of the capacitive sensors.



Tactile sensors offer valuable insights into contact forces during grasping and object manipulation. Various technologies have been utilized to create tactile sensors, including piezo-resistive, capacitive, optical, ultrasonic, piezo-electric, magnetic transduction, and barometric sensors. In a recent review by Yi et al. (Yi et al., 2018), these tactile transduction techniques were explored. One such technology, the capacitive sensor (Tiwana et al., 2011), consists of two plates separated by a gap that changes when subjected to force (see Figure 3. Capacitive sensors offer advantages such as easy integration, high sensitivity, stability, and the capability to detect both normal and tangential forces. However, they can exhibit hysteresis. Capacitive sensing is favored for capturing pressure changes due to its sensitivity, repeatability, stability, and design flexibility. It operates based on capacitance, with the two electrodes never physically touching, functioning within an elastic range, thus enhancing sensitivity and stability.

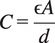

Where, 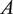 is the area of the parallel plates and 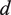 is the distance between them.

On the basis of the comparison in Table 3 among several tactile-based sensors (Pressure Profile Systems, 2024), in this work, a capacitive technology-based tactile sensor is used. FingerTPS tactile sensors, which are capacitive in nature, are utilized for recording forces exerted during the act of grasping. Positioned at the fingertips, each of these sensors is linked to a wrist-mounted module responsible for signal conditioning. This module, in turn, is interfaced with a Bluetooth transmitter for wireless communication (refer to Figure 3). The capacitance readings from the sensors are wirelessly transmitted to a computer equipped with the specialized Chameleon analysis software, through a Bluetooth receiver, facilitating real-time data analysis. To calibrate the TPS sensors and convert capacitance into force (measured in Newtons, N), each subject is instructed to complete the calibration process for all sensors at the beginning of the experimental trials. The capacitive force sensors used in this study are shielded to mitigate the impact of stray and external capacitances, which could otherwise affect sensor repeatability. This shielding ensures that external influences are minimized, maintaining consistent sensor readings and reliable measurements throughout the experiments.

**Table 3.** Tactile-based sensors comparison

Sensing technology	Capacitive	Resistive	Piezo-electric
Maximum range	Good	Good	Good
Sensitivity	Excellent	Poor	Good
Maximum element size	Good	Excellent	Poor
Repeatability	Excellent	Poor	Good
Temperature stability	Excellent	Excellent	Poor
Design flexibility	Excellent	Excellent	Good

### 2.2. Experimental setup and data acquisition

The instrumented data glove, equipped with integrated sensors, computational power, and modules for feedback on sensory performance, is proficient in detecting a firm grasp of various household objects without slippage. A specific experimental framework has been established to assess the grasping task, outlining standardized procedures that serve as a reference for evaluating the effectiveness of the measurement device.

#### 2.2.1. Objects for daily living activities (DLAs)

**Figure 4.**
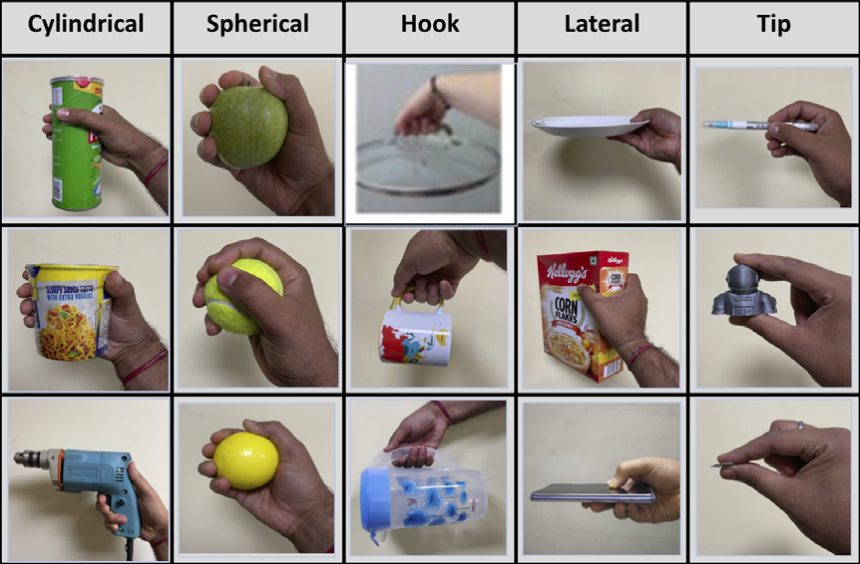
Five frequently used grasp types with three objects each used in DLAs.

Understanding the most commonly used grasp types in DLAs is crucial for assessing grasp rehabilitation processes. In this regard, the selection of grasp types is based on a combination of Schlesinger’s taxonomy (Schlesinger, 1919) and the Yale-CMU-Berkeley (YCB) set of objects (Llop-Harillo et al., 2019) that represent human grasp. These selected grasp types differentiate grasps according to the shape and size of objects typically encountered in daily activities. Figure 4 illustrates the five distinct grasp types incorporated into our proposed study: Cylindrical, Spherical, Hook, Lateral, and Tip grasp types. To provide comprehensive insights, three distinct objects are chosen for each grasp type, varying in size, mass, and stiffness. This selection strategy enables the examination of grasping attributes within the same grasp type, considering the diversity of objects. This approach aligns with prior research in grasp analysis, particularly within the domains of prostheses and rehabilitation (Feix et al., 2015).

#### 2.2.2. Subjects

Five participants were included in this study, consisting of three males and two females, with ages ranging from 25 to 45 years. All participants were right-handed and had no reported or indicated cognitive or upper extremity function complications, aligning with the criteria for individuals with hemiparesis. To be considered healthy, participants confirmed the absence of hand-related issues, such as pain, injuries, or conditions like arthritis. The experimental procedures adhered to the guidelines outlined in (Dixon, 1999), ensuring the well-being of the participants and upholding data credibility. These procedures followed established practices for human subject research. All the subjects provided written informed consent, ensuring compliance with ethical standards for research involving human subjects as per the guidelines and permission institute’s ethical committee.

#### 2.2.3. Experimental protocol

A standardized set of objects serves as a crucial foundation for achieving consistent and replicable research in grasping and manipulation. However, it is equally important to establish well-defined protocols and benchmarks that outline the experimental procedures and reliable quantification methods. In this study, data was collected during each subject session, involving multiple trial repetitions of grasping with various grasp types while wearing the glove. Participants were instructed to begin with their gloved hand in an initial resting position, palm facing downward on the table. They were then directed to grasp and lift the target object to a height of 15 cm above the table and maintain a stable hold for 15 seconds. Both sensors had a scan rate set (sampling frequency) to 40 Hz. The selected household objects had a mass below 1.5 kg, as the force sensor was calibrated to measure up to 15 newtons.

**Figure 5.**
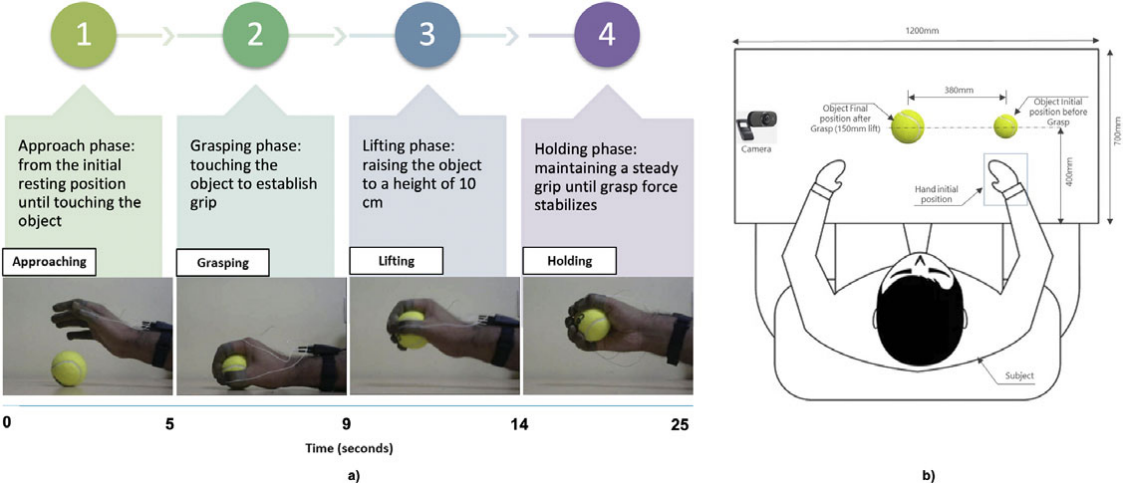
(a) Experiment timing diagram (b) experimental setup (Pratap et al., 2024).

Data was collected from each subject, involving multiple trial repetitions of grasping with various grasp types while wearing the glove. The camera was positioned at a 60 cm distance from the object to capture the grasping trials, each lasting 25 seconds. The object was placed on a table in front of the camera. As shown in Figure 5, the grasping trials were organized into four distinct phases to systematically analyze the grasping process:

1. **Approaching**: This phase extends from the initial placement of the hand until the fingers make contact with the object to be grasped.
2. **Touching for Grasping**: In this phase, the fingers make initial contact with the object, preparing for the actual grasping action.
3. **Lifting**: During this phase, participants lifted the object to a height of 10 cm above the table surface.
4. **Holding**: The final phase involved holding the object steadily, allowing time for the grasp force at each fingertip to stabilize.

These well-defined phases enabled a detailed examination of the grasping process and its associated forces. They served as a structured framework for data collection and analysis, facilitating a comprehensive understanding of hand-object interactions during grasping. During the experiment, it was observed that in some trials, the fingers made contact with the object but did not yield discernible sensor readings. This occurred when the grasp involved the finger pads and the palm, excluding the distal phalanges and fingertips where the force sensors are located. To prevent such occurrences, the experiment was conducted to ensure that grasping involved the distal phalanges of the fingers. Participants were instructed to execute their preferred natural grasps without additional constraints, resulting in a diverse range of grasp postures for the same object.

#### 2.2.4. Data collection on human grasping activity

Data acquisition was conducted across multiple subjects, employing a variety of objects and completing ten repetitions per object. The compiled dataset, formatted as .csv files, encompasses measurements of grasp force (expressed in Newtons) and the articulation of finger joints (expressed in degrees) for the thumb, index, middle, ring, and pinky fingers during each attempt. The tactile force and finger flexion records incorporate five channels, capturing data across a span of 25 seconds at a resolution of 1000 temporal increments. The temporal progression of the force and angle data was segmented into three distinct periods. Initially, for a duration of 5 seconds, the tactile force readings remain at a baseline of zero, corresponding to the subject’s approach toward the object without establishing contact. Subsequently, over the next 5 seconds, there is an escalation in force readings from nil to a peak value, delineating the phase where the subject makes contact with, grips, and elevates the object, leading to varied tactile feedback. In the final phase, lasting approximately 15 seconds, the force readings plateau, signifying a sustained and secure grasp indicative of a non-slippage condition.

## 3. Results and discussion

### 3.1. Grasp postures and forces during reach-to-grasp

Hand and finger movements during the reach-to-grasp sequence are intricately synchronized, suggesting the possibility of an overarching control mechanism guiding the grasp action in terms of posture and force. This notion of a unified control strategy aligns with the concept that the hand’s configuration progressively reveals more information as it transitions through the transport phase, culminating in the grasp where it molds to the contours of the object, as detailed by Santello (Santello et al., 1998). While the foundational idea that a limited set of synergies can define hand posture by Santello was predicated on static positions, the dynamic nature of the hand’s form during the execution of a reach-to-grasp motion needs to be explored by illustrating the evolving shape of the hand throughout this task in terms of grasp postures as well as grasp forces.

**Figure 6.**
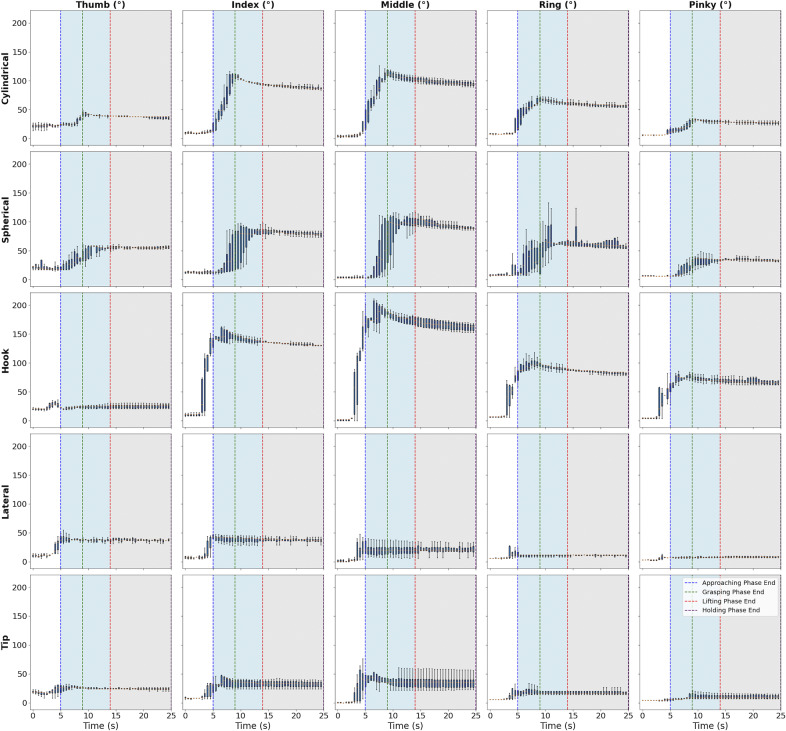
Grasp postures across the different grasp types while reach-to-grasp.

Figure 6 represents the angular motion of the fingers during various grasp types, which shows how the finger angles change according to the specific requirements of each grasp. In a typical cylindrical grasp, it is observed the thumb’s angle rapidly increases to a peak, potentially within the 60–80 degree range, within the first second, indicating an immediate and forceful closure around the object. The index and middle fingers would likely follow a similar trajectory, peaking slightly later, suggesting a sequential engagement necessary for this grip. This shows that the thumb plays a leading role in initiating the grasp, while the fingers follow in a coordinated manner to complete the grip. For a spherical grasp, the angles across all fingers would increase more gradually and peak at a lower value, implying a more distributed and encompassing movement to stabilize the object. The simultaneous yet gentler increase across the fingers indicates that the grasp is more generalized and less forceful compared to cylindrical The hook grasp would differ, with the middle and ring fingers exhibiting a slower, yet pronounced increase in angle, peaking perhaps at 100–120 degrees, reflecting a delayed engagement to hook onto the object. The delayed flexion of the middle and ring fingers highlights the specific nature of this grasp, where the fingers are gradually drawn around the object to provide a secure hold without full closure. In a lateral pinch, the thumb and index finger would display contrasting behaviors: a sharp increase in the thumb’s angle, possibly reaching 30–50 degrees quickly, versus a more moderate flexion in the index, both stabilizing as the object is pinched and lifted. This contrasting behavior emphasizes the precision needed for the lateral pinch, with the thumb providing a forceful closure while the index finger supports the object in a more controlled manner. Lastly, the tip grasp would show minimal angular variation, with a gentle peak around 10–20 degrees, resonating with the fine motor control required for delicate tasks. The minimal angular change in all fingers demonstrates the subtle and precise control needed for tasks that require delicacy. Collectively, these patterns quantitatively capture the dynamic adaptation of the human hand to the physical characteristics of objects, demonstrating a sophisticated interplay between the fingers to efficiently execute various grasping strategies.

**Figure 7.**
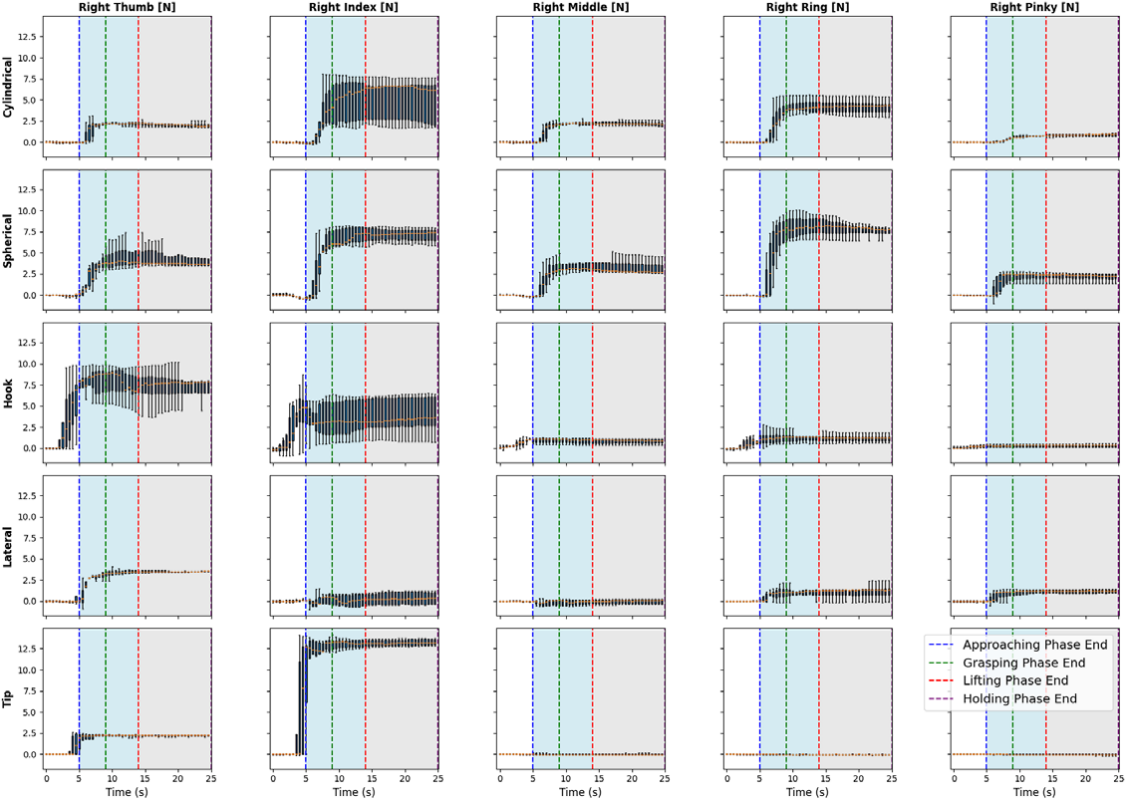
Grasp force across the different grasp types while reach-to-grasp.

Figure 7 shows the mean accuracy of grasp forces exerted by the fingers of the right hand during different grasp types over time. In the cylindrical grip, the thumb is initiating contact with a force around 2 N, surging to a peak of 10 N within 2 seconds and then holding steady at 8 N, indicative of the grip’s required strength. This pattern is echoed in the index and middle fingers, peaking at roughly 12 N and 11 N, respectively, signifying a concerted effort to secure the cylindrical object. The spherical grip presents a more balanced force distribution, with the thumb, index, and middle fingers peaking at around 7–8 N, showcasing the need to uniformly distribute force to stabilize the sphere. The hook grip highlights a later peak force in the middle and ring fingers, each reaching about 10 N by the 3-second mark, illustrating the fingers’ primary role in sustaining the object’s weight. In contrast, the lateral pinch is characterized by a swift rise in the thumb’s force to 7.5 N, against a steadier force of 5 N from the index finger, which aligns with the precision demanded by this grip type. Lastly, the tip grip demonstrates a delicate approach, with both the thumb and index finger exerting a gentle force of approximately 2.5 N, ideal for manipulating smaller items with precision. These force profiles delineated over time, shed light on the intricate mechanics of grasp and the nuanced force modulation orchestrated by the hand for object manipulation.

During the approaching phase, no significant force or change in angle is observed as the fingers are positioned for the grasp. As the grasping phase begins, there is a notable change in both angle and force, with the time to peak angle and force indicating the rapidity and strength of the grasp required. The lifting phase shows minor adjustments in angle and a maintenance or slight increase in force to secure the object against gravity. Finally, the holding phase is characterized by a stabilization of both angles and forces, maintaining the object in a steady state.

***Cylindrical Grasp Use Case:*** The results related to posture and force dynamics in the cylindrical grasp are intuitive as they align with the natural biomechanics and functional anatomy of the human hand. Each finger contributes uniquely, reflecting its structural role and physical capabilities to achieve a balanced and efficient grasp. The thumb acts as a stabilizer, countering the collective forces of the other fingers to prevent slippage, a role consistent with its versatility and strength. The index and middle fingers, positioned for precision and central stability, exert significant force and maintain controlled postures, enabling effective lateral and central support. Meanwhile, the ring and pinky fingers, with deeper flexion but lower force, provide balance and enclosure, adapting to ensure complete grip coverage and rotational stability. The synergy between posture and force is particularly evident across grasping phases—minimal force and pre-shaping in the approach phase, coordinated adjustments during the grasping phase, and force stabilization in the holding phase—mirroring the natural dynamics of daily tasks. The stabilization of forces during prolonged holding emphasizes the hand’s energy-efficient design, minimizing strain while maintaining functionality. These results intuitively reflect the hand’s ability to adapt seamlessly to grasping tasks, ensuring stability, efficiency, and precision, consistent with its remarkable biomechanical design.

This analysis indicates a highly coordinated control strategy, where the fingers pre-shape and prepare for object interaction, swiftly adjust to grasp the object, maintain grip against gravity, and hold the object securely, reflecting the adaptability and precision of the human hand.

### 3.2. Cross-sectional analysis

To initiate the design process of a generic hand exoskeleton for hand assistance, a cross-sectional study of fingertip forces was performed across two distinct situations. This investigation involves calculating the Mean Absolute Deviation ( 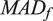 ) for the force exerted by each finger. 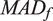 is utilized as an indicator of variation, evaluating the average deviation of each force measurement from the overall average. The equation for 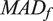 is presented as:

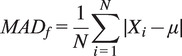



Here, *Xi* represents the individual force values, 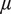 denotes the mean of these values, and *N* indicates the total number of force values associated with each finger. This calculation allows us to gauge the variations in force values for specific fingers concerning changes in subjects and objects. It provides insights into the range of force values exerted by a particular finger when grasping objects typically encountered in Activities of Daily Living (DLAs).

**Figure 8.**
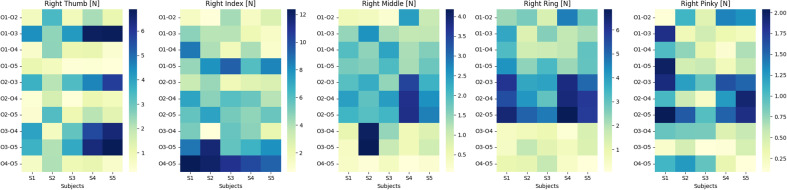
Mean absolute deviation across subjects.

**Figure 9.**
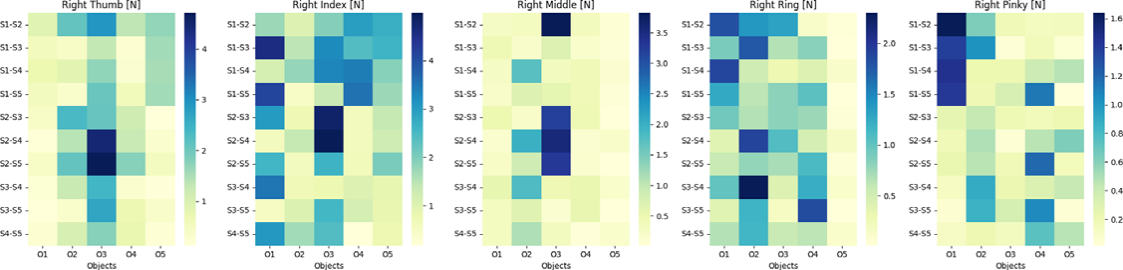
Mean absolute deviation across objects.

Furthermore, to enhance the understanding of 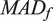 , we present graphical illustrations (refer to Figures 8 and 9) for pairs of fingers under varying subject and object conditions in two separate cases. In the analysis section, the term “object” is used to represent specific grasp types, which are denoted as O1, O2, O3, O4, and O5. These codes correspond to the Cylindrical, Spherical, Hook, Lateral, and Tip grasp types, respectively. For each grasp type, these visual representations help visualize the variations and trends in 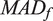 across different scenarios.

#### Case I: Varying objects keeping the subject the same

This case of our cross-sectional study presents a quantitative analysis of the mean absolute difference (MAD) in force values exerted by the right hand’s fingers, this time focusing on the variation across different objects while keeping the subject constant. In the provided heat maps (Figure 8), we observe the following patterns for each fingertip. The highest MAD value observed is 6.9 N between objects O3 and O5 for subject S3, indicating substantial variability in thumb force when handling different objects. This contrasts with a lower variability of 0.22 N between objects O1 and O5 for subject S1, suggesting that the thumb’s force application is more consistent with these particular objects. A notable MAD value of 12 N is present between objects O4 and O5 for subject S1, which is the maximum across all fingers and indicates a pronounced difference in index finger force exertion between these objects. On the lower end, a MAD of 0.31 N between objects O4 and O5 for subject S5 reflects minimal variability. The highest MAD of 3.9 N is evident between objects O2 and O3 for subject S4, pointing to a considerable variation in the force exerted by the middle finger across these objects. The lowest variability is observed as a MAD of 0.19 N between objects O4 and O5 for subject S5. The MAD peaks at 6 N between objects O2 and O3 for subject S3, suggesting a significant disparity in force exertion on different objects by the ring finger. Conversely, a lower MAD value of 0.47 N is noted between objects O4 and O5 for subject S4, indicating a consistent force application across these objects. For the pinky finger, the highest MAD is recorded at 2.9 N between objects O1 and O2 for subject S1, illustrating considerable variability in force exertion. Meanwhile, a minimal MAD of 0.088 N between objects O4 and O5 for subject S5 denotes a high degree of consistency in force application.

These findings underscore the influence of object characteristics on grasp force variability, emphasizing the need for a hand exoskeleton design that is capable of accommodating a broad spectrum of object-induced force variations. The data suggests that the exoskeleton should provide tailored assistance that adapts to the diverse demands placed on each finger by different objects, ensuring that the device supports the user effectively across the full range of DLAs.

#### Case II: Varying subjects keeping the object the same

This case of our cross-sectional study presents a quantitative analysis of the MAD in force values exerted by the fingers, this time focusing on the variation across different subjects while keeping the object constant. We employed heat maps (Figure 9) to quantify the MAD in force values across five fingers under varying conditions. For the thumb, the MAD peaked at 4.4 N between subjects S2 and S4, suggesting a substantial disparity in the applied force during object manipulation. Conversely, the lowest variability was observed at 0.13 N between subjects S3 and S5. The index finger exhibited a similar pattern of variability, with the highest MAD recorded at 4.8 N between subjects S2 and S3, indicating significant individual differences in force application. In the case of the middle finger, the MAD values ranged considerably, with the highest at 3.8 N between subjects S1 and S3. For the middle finger, the MAD values exhibited notable variation, with the highest recorded at 3.8 N between subjects S1 and S3, emphasizing the diversity in force exertion for this digit among subjects. The ring finger’s maximum MAD was 6 N between subjects O2 and O3 when interacting with different objects, highlighting the influence of object characteristics on force application. Lastly, the pinky showed a prominent MAD value of 2.9 N between subjects O1 and O2, reinforcing the significance of individual differences in finger strength and dexterity. These quantitative findings offer a critical foundation for the design of a hand exoskeleton, necessitating a versatile and responsive system that can adapt to the varied force profiles encountered in DLAs.

**Figure 10.**
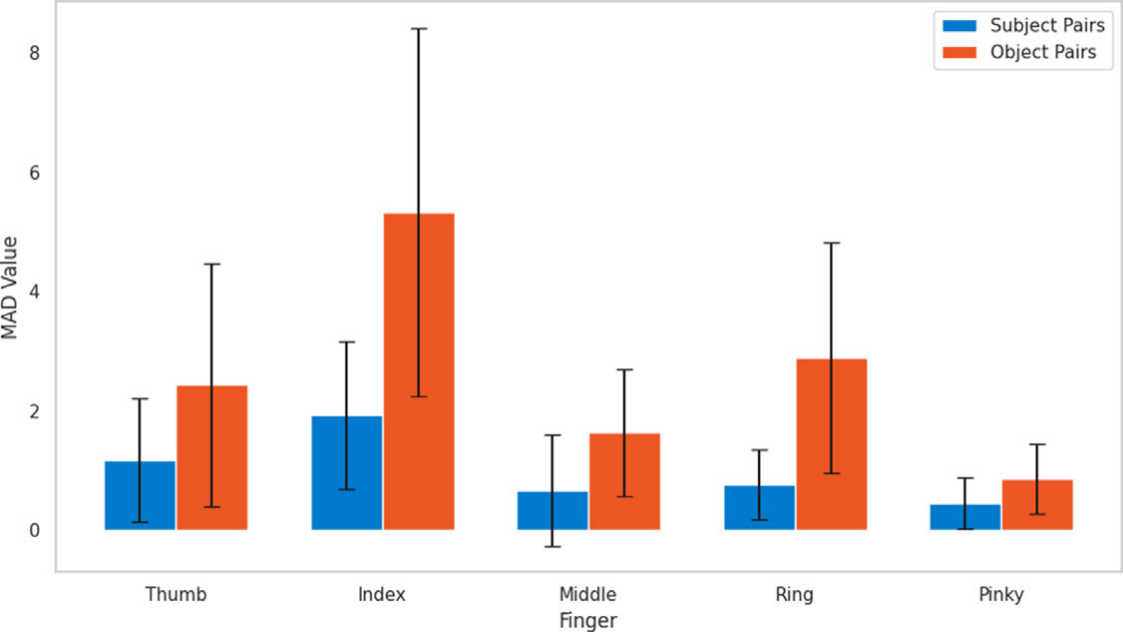
Comparison of mean absolute deviation values across subject and object pairs.

The provided bar chart in Figure 10 and the statistics in Table 4 delineate the Mean Absolute Difference (MAD) in force values across subject and object pairs for each finger, offering a clear visual and quantitative comparison. For the thumb, object pairs exhibited a higher mean MAD (2.439 N) with a substantial maximum MAD (6.883 N), compared to subject pairs which had a lower mean (1.172 N) and maximum MAD (4.704 N). The index finger showed even greater variability for object pairs, with a mean MAD of 5.341 N and a maximum of 12.356 N, significantly exceeding the mean (1.928 N) and maximum (4.979 N) for subject pairs. The middle finger’s force variability was more moderate, with object pairs having a mean MAD of 1.642 N and a maximum of 4.175 N, while subject pairs had a mean of 0.672 N and a maximum of 3.817 N. The ring finger’s object pair variability was also notable with a mean MAD of 2.890 N and a maximum of 6.850 N, in contrast to the subject pairs’ mean of 0.761 N and maximum of 2.297 N. Lastly, the pinky finger, while showing a least variability, still had a higher mean MAD for object pairs (0.863 N) with a maximum of 2.038 N, against the subject pairs’ mean of 0.452 N and maximum of 1.638 N. These statistics underscore the pronounced effect of object characteristics on force exertion compared to the variability between subjects, highlighting the need for an adaptable exoskeleton design to cater to these differences, as illustrated by the significant standard deviations observed for object pairs across all fingers.

**Table 4.** Combined statistics of MAD for subject and object pairs

	Subject pairs					Object pairs				
	Thumb	Index	Middle	Ring	Pinky	Thumb	Index	Middle	Ring	Pinky
max	4.70	4.98	3.82	2.30	1.64	6.88	12.36	4.17	6.85	2.04
min	0.13	0.18	0.02	0.01	0.02	0.22	0.62	0.06	0.07	0.09
mean	1.17	1.93	0.67	0.76	0.45	2.44	5.34	1.64	2.89	0.86
median	0.87	1.71	0.33	0.66	0.25	1.80	4.76	1.53	2.50	0.80
std	1.04	1.24	0.94	0.58	0.43	2.04	3.09	1.06	1.93	0.58

***Cylindrical Grasp Use Case:*** To explain the intuitive nature of the results, let us consider the cylindrical grasp. Cylindrical objects naturally conform to the shape of the hand, allowing for a uniform distribution of forces across the fingers. The low 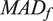 values for varying objects indicate consistent force application across different cylindrical objects, as the shape provides a predictable surface area for the hand. Minor variations in object properties, like texture or weight, may cause slight deviations in force, but the grasp strategy remains stable. In the case of varying subjects, the higher 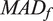 values reflect individual differences in hand anatomy, strength, and motor control. Larger hands distribute forces more evenly, while smaller hands may rely more on specific fingers. Similarly, subjects with greater grip strength apply less force due to efficiency, whereas those with weaker hands compensate by exerting higher force. These variations emphasize the need for an exoskeleton that accommodates individual differences. Overall, the results align with the intuitive understanding of hand biomechanics, offering valuable insights into designing adaptable assistive devices.

### 3.3. Grasp synergy- radar plots

**Figure 11.**
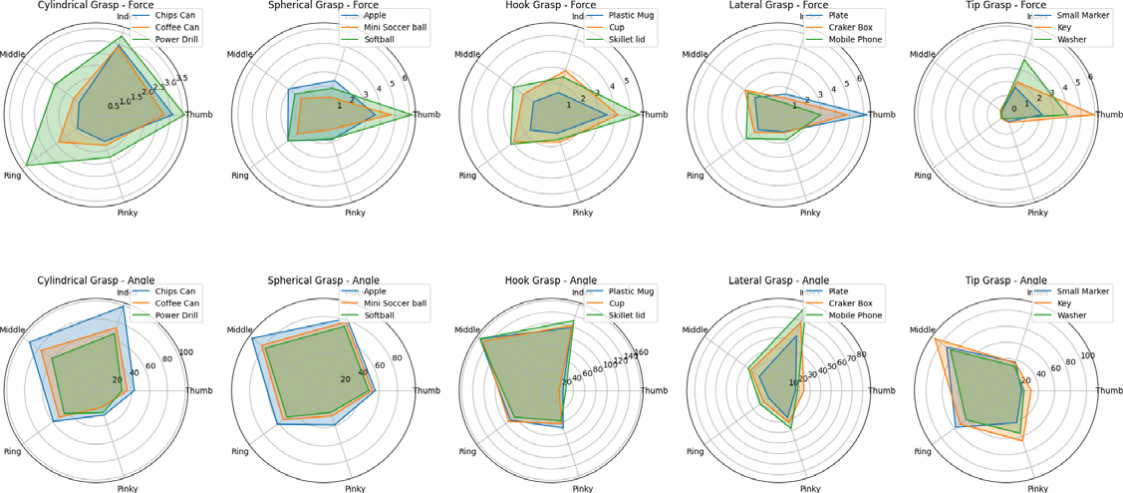
Radar plots for various grasp types across all the objects in terms of force and postures.

Radar (or spider) charts, which are graphical methods to display multivariate data in the form of a two-dimensional chart of three or more quantitative variables represented on axes starting from the same point. Figure 11 shows a comprehensive visualization of hand grasp synergies, quantifying both the force applied by each finger during various grasps and the corresponding finger bending angles. Each chart represents different types of grasps: Tip Grasp, Spherical Grasp, Hook Grasp, Lateral Grasp, and Cylindrical Grasp. The charts are divided into two categories for each grasp type: Force and Angle. These compare the finger bending angles (postures) and grasp forces applied by each finger (Thumb, Index, Middle, Ring, Pinky) when grasping various objects. The objects for each grasp type are listed in the legends. Each axis or spoke on these charts (vertex of the pentagon) corresponds to a specific finger and grasp combination, with the length of the spoke indicating the average magnitude of force or angle observed across all subjects and trials.

In Figure 11, consistent geometrical patterns emerge across the pentagonal charts, illustrating the collective action and coordination of the fingers in exerting force and achieving the required posture for each grasp type. The analysis reveals that objects of greater mass are associated with larger areas within the chart, indicating a direct correlation between an object’s mass and the necessary grasp force. Conversely, variations in the cross-sectional area of the objects are inversely related to the bending angles, with bulkier objects leading to a smaller enclosed area in the chart, signifying a reduction in the required bending angles for grip stability.

In the cylindrical grasp, the thumb’s force is paramount, peaking at 3.5 units for the power drill, indicating its key role in opposition and stabilization. Correspondingly, wider angles between the thumb and index finger are noted, particularly evident with the power drill, suggesting a significant wraparound grasp necessary for a secure hold. This is intuitive because the thumb must oppose the object to stabilize the grasp, while the moderate bending angles of the fingers ensure efficient force distribution without compromising stability. The radar plots also show a more enclosed area for the power drill, intuitively indicating less need for extreme bending angles to maintain a stable grasp. For the spherical grasp, the force distribution is more uniform across the fingers, yet the thumb is notably engaged, applying approximately 5 units of force when holding a softball. This demonstrates the thumb’s role in enveloping and counterbalancing the spherical object’s form. Angles for all fingers are larger, with the thumb exceeding 60 degrees, which is required to maintain contact with the object’s curved surface. The hook grasp shows a moderate force profile across the fingers, with the thumb slightly more engaged, indicative of its role in counterbalancing the hooking action exerted by the other fingers. A substantial thumb flexion is also observed, reaching up to 160 degrees, necessary for securing objects from above. In the lateral grasp, the thumb’s force is again pronounced, especially critical for pressing against the flat sides of objects like a mobile phone. This is complemented by the thumb’s acute flexion, reflecting its dynamic role in pinching movements. The tip grasp reveals the index finger’s dominance in precision tasks, exerting the most force, slightly above 4 units for small objects such as a key. This precision is also mirrored in the joint angles, with smaller angles observed for the thumb and index finger, allowing for detailed manipulation of diminutive items.

These charts serve as a tool for analyzing the dynamic and kinematic coordination of the hand, revealing the complex interplay of forces and movements required for diverse grasping actions. Overall, these results align with natural hand biomechanics, where force and posture are coordinated to optimize grasp stability and control, making them highly intuitive for both human and robotic grasp design. The significance of these findings lies in their application to the design of robotic hands and prosthetics. By understanding the force distribution and joint angles necessary for different grasp types, engineers can create more nuanced and effective grasp controllers. These controllers would need to replicate the varied force application and joint angles observed in human hands, ensuring that robotic or prosthetic devices can perform a wide range of tasks with the same dexterity as a human hand. This study’s grasp synergy analysis is critical for achieving such biomimetic functionality.

### 3.4. Quantitative analysis of finger force-angle correlations in grasping

To delve deeper into the intricacies of hand movements and force application during the act of grasping, we investigate the correlation between the forces exerted and the angular positions of the fingers, as recorded by the data glove. The gathered dataset, which encompasses both angular and force data for each finger across a range of grasping actions involving the five distinct grasp types, serves as the foundation for this analysis. Identifying similarities in force application patterns and probing the association between the forces at the fingertips and the bending angles of the fingers during a grasp is crucial for discerning synergistic movements. Our detailed examination has revealed significant associations across the fingers, considering the forces and angles over multiple objects, subjects and trials. These associations are quantified through the Pearson Correlation Coefficient, which provides a statistical measure of the strength of a linear relationship between paired data. The coefficient, denoted as r, can be computed by the following formula:

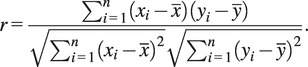



**Figure 12.**
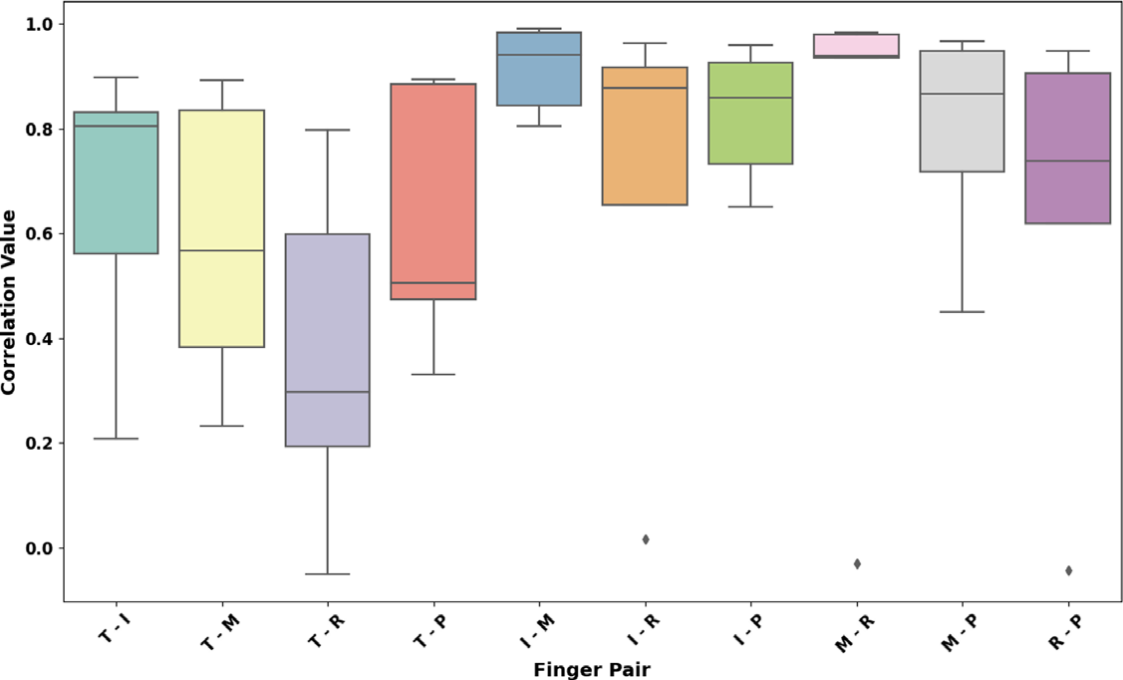
Correlation coefficients for grasp postures between finger pairs.

**Figure 13.**
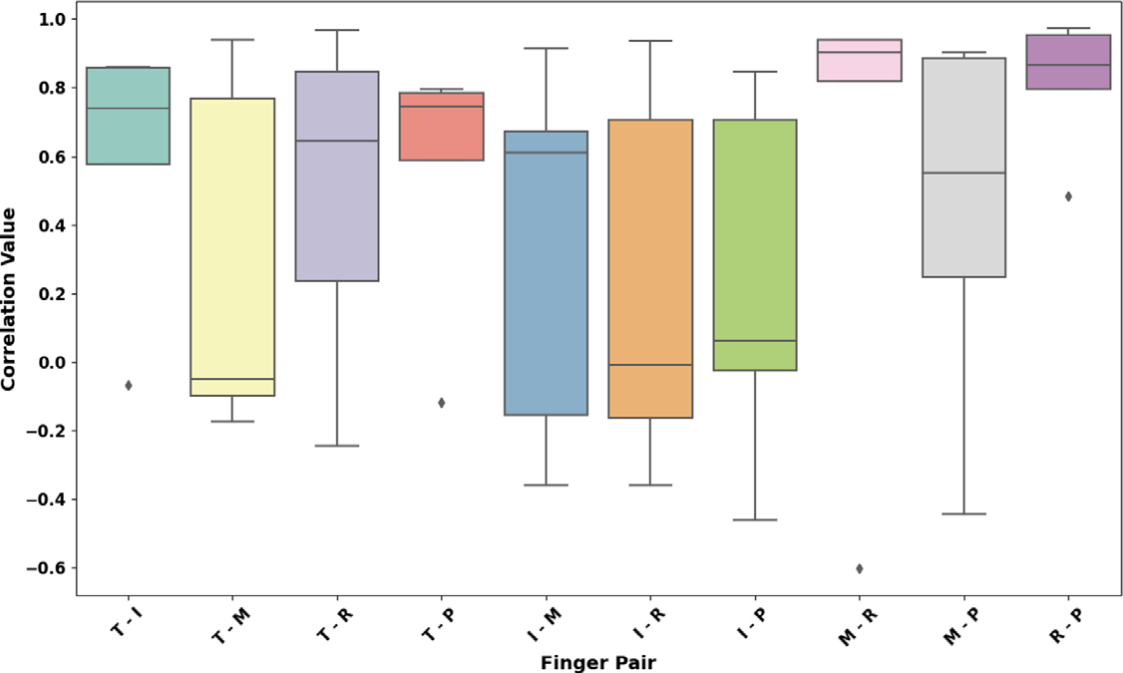
Correlation coefficients for grasp forces between finger pairs.

Table 5 lists the overall correlation coefficients for different finger pairs across various grasp types, quantifying the relationship between grasp posture and force. The box plot in Figures 12 and 13 portrays the distribution of correlation coefficients for different finger pairs with respect to their grasp postures and forces. The correlation coefficient is a statistical measure ranging from 0 to 1, where values closer to 1 indicate a stronger positive association between the two fingers during grasping. From left to right, the finger pairs are denoted as T-I (Thumb-Index), T-M (Thumb-Middle), T-R (Thumb-Ring), T–P (Thumb-Pinky), I-M (Index-Middle), I-R (Index-Ring), I-P (Index-Pinky), M-R (Middle-Ring), M-P (Middle-Pinky), and R-P (Ring-Pinky). Quantitatively, the median values of correlation coefficients suggest varying degrees of coordination between different finger pairs.

**Table 5.** Grasp posture and force correlation coefficients for finger pairs and objects

	Cylindrical		Spherical		Hook		Lateral		Tip	
Finger Pair	Posture	Force	Posture	Force	Posture	Force	Posture	Force	Posture	Force
T-I	0.83	0.74	0.90	0.86	0.21	0.58	0.81	−0.07	0.56	0.86
T-M	0.84	0.94	0.89	0.77	0.23	−0.17	0.38	−0.05	0.57	−0.10
T-R	0.80	0.97	−0.05	0.85	0.30	0.24	0.19	0.64	0.60	−0.24
T–P	0.89	0.74	0.89	0.80	0.33	0.59	0.51	0.78	0.47	−0.12
I-M	0.94	0.61	0.98	0.91	0.99	−0.36	0.80	0.67	0.84	−0.15
I-R	0.92	0.70	0.02	0.94	0.96	−0.01	0.65	−0.36	0.88	−0.16
I-P	0.93	0.70	0.96	0.85	0.86	0.06	0.73	−0.46	0.65	−0.02
M-R	0.98	0.94	−0.03	0.94	0.98	0.82	0.94	−0.60	0.93	0.90
M-P	0.95	0.55	0.97	0.88	087	0.25	0.72	−0.44	0.45	0.90
R-P	0.95	0.79	−0.04	0.97	0.91	0.48	0.74	0.87	0.62	0.95

According to Figure 12, the T-I pair, with a median correlation nearing 0.8 and a narrow interquartile range, reflects a strong and consistent synergy, indicative of their collaborative role in precision tasks like writing or buttoning. The T-M pair, though exhibiting a slightly lower median correlation above 0.6, still shows a significant degree of coordination, albeit with more variability potentially due to the middle finger’s dual function in precision and power grips. T-R and T–P pairs present median correlations around 0.5, with broader spreads suggesting less consistency in their cooperative movements, aligning with the more supportive roles these fingers play in grip stability. The I-M pair, with a correlation akin to the T-I pair, underscores a robust synergy essential for the manipulation of objects, while the I-R and I-P pairs, with correlations just over 0.6, suggest moderate coordination, possibly due to the stabilizing influence of the ring and pinky fingers. The M-R and M-P pairs show lower median correlations, reflecting a tendency for independent movement, consistent with the less central role these fingers have in many grasping actions. Conversely, the R-P pair’s median correlation suggests that, despite being supporting digits, they frequently act in concert, particularly in power grips where they contribute to the envelopment and stabilization of objects. This investigation into the coordinated actions of finger pairs during grasping reveals essential patterns that have profound applications in the realm of prosthetic design and robotics. By mapping the nuanced relationships between finger movements, designers can create prosthetic hands that mimic the intricate and natural motions of the human hand, enhancing their functionality and utility.

The box plot (Figure 13) depicting correlation coefficients for grasp forces among finger pairs provides a quantitative insight into the synchronous force application during various grasp types. The T-I pair, with a median correlation just below 0.8, and the I-M, nearly at 0.9, demonstrate strong cooperative force dynamics, pivotal for precision handling. The T-M pair, showing a median nearing 0.9, suggests a highly synergistic interaction essential for manipulating and securing objects. Conversely, the T-R and T–P pairs, with median correlations around 0.6 and just over 0.5 respectively, exhibit more moderate coordination, aligning with their roles in providing grasp stability rather than manipulation. The I-R and I-P pairs, both hovering around a median of 0.7, suggest a significant yet less pronounced force relationship, likely engaged in diverse grasping actions. The M-R correlation, just below 0.8, and the R-P, close to 0.8, reveal a strong link, emphasizing their collective function in maintaining a secure and balanced grip. The M-P pair, with a median correlation around 0.7, indicates substantial synergy, which is critical in grips that require enveloping an object.

***Cylindrical Grasp Use Case:*** The results related to pose and force in the cylindrical grasp type are highly intuitive when considering the natural biomechanics of the human hand. In cylindrical grasps, the thumb plays a crucial role in providing stability and opposing the object, which is evident in the strong correlations observed between the (T-I) and T-M pairs. For example, the T-I pair shows a high correlation, indicating a strong and consistent synergy between these fingers, which aligns with their collaborative function in holding and stabilizing cylindrical objects such as a cup. The thumb’s role in applying force to stabilize the object is reflected in the median correlation for grasp forces between the Thumb-Index pair, which is just below 0.8. Additionally, the middle finger contributes to the precision and strength of the grip, as seen in the moderate correlation between the T-M and M-R pairs. The broader range in the T-R and T–P pair correlations reflects their more secondary roles in supporting the object, highlighting the thumb’s central role in gripping. The forces applied by the thumb and index fingers are tightly coordinated, as seen in the high correlation coefficient between their force application, while the pinky and ring fingers contribute less directly to force generation but help in stabilizing the object. These force patterns, combined with the angular relationships between fingers, match the expected natural behavior when grasping cylindrical objects, with fingers working in harmony to achieve a stable and functional grip. This alignment of force and posture correlations with the natural hand function reinforces the intuitive nature of the results and provides insight into how human hand biomechanics can be replicated or enhanced in five-fingered robotic systems. This comprehensive correlation assessment across finger pairs is invaluable for advancing exoskeleton/prosthetic design, enhancing robotic gripper functionality, and optimizing rehabilitation techniques for hand function recovery, aiming to replicate the intricate force patterns and cooperative dynamics of the human hand.

## 4. Conclusion

Our comprehensive study has yielded data on human hand grasping, quantifying the interplay of forces and postures that underlie the natural redundancy of the human hand. This redundancy, often considered a hallmark of human dexterity, is not only reflected in the ability of fingers to compensate for each other but also in the distinct yet complementary roles played by each digit in various grasp types. The employment of a multisensory data glove has proven instrumental in capturing the nuanced synergies between force and posture. Our findings indicate a significant degree of collaboration between finger movements across various grasps and conditions, suggesting that each finger pair contributes distinctly to the overall grasp pattern. The correlation analysis between finger pairs has provided valuable insights into the cooperative behavior crucial for effective hand function, underscoring the intricate redundancy mechanisms that ensure robust grasping even under varying conditions. These results underscore the importance of considering individual variability when designing assistive devices and rehabilitation tools, as the one-size-fits-all approach is insufficient for accommodating the diverse needs of users.

Moreover, the use of spider charts to visually represent grasp synergies has highlighted the unique characteristics of different grasp types, affirming the potential of visual tools in enhancing our understanding of complex biomechanical data. This study advances the grasp synergy discourse by not only confirming well-known principles, such as the opposing thumb’s pivotal role but also quantifying how individual finger contributions vary across grasp types and conditions. These quantifications reveal the adaptability of grasp patterns and the nuanced interplay of redundancy, force coordination, and posture, which are essential for effective manipulation. In conclusion, our research contributes significantly to the grasp synergy discourse, offering a foundation for future innovations in underactuated device design and personalized rehabilitation strategies. It opens up new avenues for the creation of exoskeletons, prosthetics and robotic hands that can mimic the intricate coordination and force application of the human hand. By leveraging the quantified understanding of hand redundancy and synergies, we can design five-fingered robotic hands or exoskeletons that not only replicate but also adapt to the complex dynamics of human grasping, offering more robust and versatile solutions. The primary objective of this research is to analyze the kinematic and kinetic correlations among the fingers during various grasp types using sensor-based data. While the study provides valuable insights into finger synergies, its focus on data analysis limits the inclusion of detailed mechanical modeling and simulations of contact forces. By translating these synergistic patterns into the mechanics of five-fingered robotic devices, we can strive towards more intuitive and effective solutions that enhance the quality of life for individuals requiring assistance with hand functions. As we move forward, the implications of this study will undoubtedly resonate within the fields of biomedical engineering, rehabilitation, and human-computer interaction, driving forward the development of technologies that are deeply in sync with the natural capabilities of the human body. The study’s implications extend to the design of flexible, 3D-printed data gloves, promising advancements in the integration of such technologies into daily life and medical practice. Additionally, a critical next step is translating the quantified grasp synergies into practical applications, such as robotic hands or task-specific prosthetics, which will significantly extend the impact of this research on real-world assistive technologies.

## Data Availability

Data can be made available to interested researchers upon request by email to the corresponding author.
